# A limited number of double-strand DNA breaks is sufficient to delay cell cycle progression

**DOI:** 10.1093/nar/gky786

**Published:** 2018-09-03

**Authors:** Jeroen van den Berg, Anna G. Manjón, Karoline Kielbassa, Femke M Feringa, Raimundo Freire, René H Medema

**Affiliations:** 1 Oncode Institute, Division of Cell Biology, The Netherlands Cancer Institute, Plesmanlaan 121, 1066 CX Amsterdam, The Netherlands; 2 Unidad de Investigación, Hospital Universitario de Canarias, Instituto de Tecnologías Biomédicas, Ofra s/n, La Laguna, Tenerife, Spain

## Abstract

DNA damaging agents cause a variety of lesions, of which DNA double-strand breaks (DSBs) are the most genotoxic. Unbiased approaches aimed at investigating the relationship between the number of DSBs and outcome of the DNA damage response have been challenging due to the random nature in which damage is induced by classical DNA damaging agents. Here, we describe a CRISPR/Cas9-based system that permits us to efficiently introduce DSBs at defined sites in the genome. Using this system, we show that a guide RNA targeting only a single site in the human genome can trigger a checkpoint response that is potent enough to delay cell cycle progression. Abrogation of this checkpoint leads to DNA breaks in mitosis which gives rise to aneuploid progeny.

## INTRODUCTION

As much as 10,000 DNA lesions arise in a human cell per day, most of which are caused by oxidative damage (1,2). Proper management and repair of these DNA lesions is essential for development and tissue homeostasis and helps avert tumorigenesis (3–6). Most crucial to cell viability are the pathways involved in double-strand breaks (DSBs) responses, as these represent the most genotoxic lesions (1,7). Historically, studies aimed at a better understanding of DNA damage control have centered on the discovery of genes involved in sensitivity to DNA damaging agents (1,8). These studies have led to the identification of a variety of damage repair pathways that act to detect and repair DNA damage. It is currently still largely unclear how these pathways act together in different genomic locations and how they are influenced by chromatin context (9,10).

Recent observations have sparked an interest in the influence of distinct chromatin states on the execution of DNA damage responses (11). Classic experimental approaches such as the use of DNA damaging agents like Topoisomerase II poisons or γ-irradiation induce breaks at random locations in the genome, making them unsuitable as tools to study site specific DSBs. Initial evidence supporting the hypothesis that local chromatin state can influence DNA damage responses has therefore come from studies using selective endonucleases, which are able to generate DSBs at single or multiple sites (12–15). Although selective endonucleases have given us some insights regarding location-dependent effects on DNA damage responses, their applicability for unbiased investigations are limited due to a minimal regiment of target-sites in the genome (i.e. I-PpoI) or the requirement to introduce a *de novo* restriction site in the genome (i.e. I-SceI). Current advances in genome engineering allow us for the first time to target many, if not all, loci without the need for the introduction of de-novo sequences in the genome (16). The genome editing technique that is currently most used is Type II clustered regularly interspaced short palindromic repeats (CRISPR), originating from a bacterial adaptive immune system that introduces DSBs in the genome of bacteriophages, thereby perturbing their bacterial virulence (17,18).

Previous work from our lab and others has shown that CRISPR can be used to tease apart location-dependent effects on checkpoints and cell fate decisions, but the systems that were used for these studies lacked sufficient temporal control over break formation (19–21). Here, we report the generation of a time-controlled Cas9 system that allows us to induce a defined number of DSBs at very specific sites in the genome and subsequently monitor repair and cell fate. This system allows us to address how number and location of breaks influence the overall DNA damage response (DDR) and checkpoint activation.

Here we show, by using a tractable Cas9 system, that a limited number of DSBs is sensed by the DNA damage checkpoint and can delay cell cycle progression.

## MATERIALS AND METHODS

### Antibody generation

Anti-Cas9 was raised against the first 300 amino acids of Cas9 from *Streptococcus pyogenes*. The cDNA corresponding to amino acids 1–300 of Cas9 from *S. Pyogenes* was cloned in pET-30a (Novagen). The resulting 6x His tagged antigen was expressed in *Escherichia coli*, purified and used for rabbit immunization. Rabbit polyclonal antiserum was affinity purified.

### Plasmid construction

pCW-Cas9 was a gift from Eric Lander and David Sabatini (Addgene plasmid # 50661). The iCut plasmid was generated by linearizing pCW-Cas9 with NheI and introducing the FKBP destabilization domain. The FKBP destabilization domain was amplified from the pRetroX-Tight FKBP-I-PpoI plasmid (20) using primers suitable for Gibson Assembly (22).

### Cell lines, tissue culture and irradiation

Retinal pigment epithelial (RPE-1) hTERT cell lines (obtained from American Type Culture Collection) were maintained in DMEM/F12 GlutaMAX medium (Gibco) containing 10% Tetracycline-free Fetal Bovine Serum and 1% Penicillin-Streptomycin (10 000 U/ml). DiC-RPE-1 cells were generated by lentiviral transduction with first generation lentiviral helper plasmids of pCW-Cas9 (23). iCut-RPE-1 cells were generated by transduction of the iCut plasmid, followed by selection with Puromycin (20 μg/ml). Constitutive Cas9 expressing cells were previously described (20). Chemicals used in this study: Doxycycline (Sigma, 1 mM), SHIELD-1 (Aobious, 1 μM), Nutlin-3a (Cayman Chemical, 10 μM), Wee1 inhibitor MK-1775 (3 mM, Sigma), ATM inhibitor Ku55933 (10 μM, Merck-Millipore), ATR inhibitor VE-821 (10 μM, Selleck) and Nocodazole (250 μM, Sigma). Cells were γ-irradiated using a Gammacell Exactor (Best Theratronics) with a ^137^Cs source.

### tracrRNA:crRNA design and transfections

Alt-R crRNA (Integrated DNA technologies) were designed with on-target scores determined by the Rule Set 2 (24). Off-target scores were determined by the specificity score (25). For HS1, we used the previously described crRNA targeting the *LBR* gene (26). For HS4, we used a sequence of the *NF2* gene and processed similarly as HS13 and HS18 to select a crRNA with the most target sites. For HS13, HS15 and HS17; we used *RPL12* pseudogenes to design sgRNAs with the rationale that these would target multiple sequences. We used the *RPL12P38* pseudo-gene annotated in the hg19 assembly of the human genome. Subsequently, we selected sgRNAs based on the CRISPOR (27). We included predicted sites with complete homology and with maximum 1 mismatch outside of the seed sequence of the sgRNA (position 1–8 (28)). Out of all the targets none target coding sequences of genes. tracrRNA:crRNA duplex was transfected according to manufacturer's protocol (29).

The following crRNA were used in this study:

**Table:** 

eGFP 5′-GTCGCCCTCGAACTTCACCT-3′	Doench 2016 [70], Hsu 2013 [81]
*TP53* 5′-TCGACGCTAGGATCTGACTG-3′	Doench 2016 [64], Hsu 2013 [48]
HS1 5′-GCCGATGGTGAAGTGGTAAG-3′	Doench 2016 [73], Hsu 2013 [55]
HS4 5′-TGGACTGCAGTACACAATCA-3′	Doench 2016 [58], Hsu 2013 [16]
HS13 5′-AGAAAAACATTAAACACAGT-3′	Doench 2016 [58], Hsu 2013 [6]
HS15 5′-TTTTTGGAGACAGACCCAGG-3′	Doench 2016 [77], Hsu 2013 [5]
HS17 5′-CAGACAGGCCCAGATTGAGG-3′	Doench 2016 [70], Hsu 2013 [4]

### Clonogenic assays

iCut-RPE-1 or DiC-RPE-1 cells were transfected with the indicated crRNAs and 16 h later, 250 single cells per well were seeded in six-well plates. Cells were treated with the indicated drugs and allowed to grow out for 7 days. Plates were fixed in 80% ice-cold Methanol and stained with 0.2% Crystal Violet solution. Colonies were counted and normalized to plating efficiency of untreated control.

### Determination of insertions and deletion by TIDE and LM-PCR

Insertions and deletion of the *TP53* locus were quantified by PCR amplification of the edited region with the following primer set (5′- GGGAAGGTTGGAAGTCCCTCTC -3′ and GCTTCATCTGGACCTGGGTCTT). For the HS1 locus, genomic DNA was subjected to PCR with the following primer set (5′ GTAGCCTTTCTGGCCCTAAAAT 3′ and 5′ AAATGGCTGTCTTTCCCAGTAA 3′) The PCR products were subjected to Sanger Sequencing and analyzed by the Tracking of Indels by Decomposition (TIDE) method (26). Ligation mediated PCRs were performed as previously described (30).

### Fixed cell microscopy

Images were obtained using a DeltaVision Elite (Applied Precision) maintained at 37°C equipped with a 60 × 1.45 numerical aperture (NA) lens (Olympus) and cooled CoolSnap CCD camera. DNA damage foci were evaluated in ImageJ, using an in-house developed macro that enabled automatic and objective analysis. In brief, cell nuclei were detected by thresholding on the (median-filtered) DAPI signal, after which touching nuclei were separated by a watershed operation. The foci signal was background-subtracted using a Difference of Gaussians filter. For every nucleus, foci were identified as regions of adjacent pixels satisfying the following criteria: (i) the gray value exceeds the nuclear background signal by a set number of times (typically 2-fold) the median background standard deviation of all nuclei in the image, and is higher than a user-defined absolute minimum value [1]; (ii) the area is larger than a defined area (typically 16 pixels). These parameters were optimized for every experiment by manually comparing the detected foci with the original signal.

### Live-cell imaging

Cells were grown in Lab-Tek II chambered coverglass (Thermo Scientific) in Dulbecco's Modified Eagle Medium/ Nutrient Mixture F-12 (DMEM/F12) outfitted with a CO2 controller set at 5%. Images were obtained using a DeltaVision Elite (Applied Precision) maintained at 37°C equipped with a 10× or 20× PLAN Apo S lens (Olympus) and cooled CoolSnap CCD camera. Macros used to quantify foci living cells were previously described (31). FUCCI experiments were performed and analyzed as previously described (32).

### FACS analysis

For G2 checkpoint recovery, we synchronized cells at the G1/S transition using a thymidine block, we induced Cas9-activity by the addition of doxycycline/SHIELD-1 at the time of thymidine addition. At the time of release, the cells were transfected with indicated gRNAs and the cells were allowed to progress into G2. Nocodazole was added 7 h post transfection to trap cells in mitosis over a period of 16 h. Cumulative mitotic entry was determined by the percentage of MPM-2-positive cells (33). For H2B-eGFP disruption, we fixed cells in 70% ethanol 4 days after the washout of the agonists (doxycycline for DiC, doxycycline, and SHIELD-1 for iCut). Disruption was analyzed by determining the percentage eGFP negative cells in the total population.

### Immunofluorescence and Western Blots

For IF, cells were fixed with 3.7% formaldehyde for 5 min and permeabilized with 0.2% Triton-X100 for 5 min before blocking in 3% bovine serum albumin (BSA) in PBS supplemented with 0.1% Tween (PBS-T) for 1 h. Cells were incubated overnight at 4°C with primary antibody in PBS-T with 3% BSA, washed three times with PBS-T, and incubated with secondary antibody and DAPI in PBS-T with 3% BSA for 2 h at room temperature (RT). Western blot analysis was performed as previously described (31). The following primary antibodies were used in this study: anti-Cas9 (homemade rabbit polyclonal, 1:2000), anti-MPM2 (05-368, Millipore, 1:500), anti-γH2AX (ser139p; 05-636 Upstate, 1:500), anti-53BP1 (H-300, Santa Cruz, sc-22760, 1:500), anti-Cdk4 (C-22; sc-260 Santa Cruz, 1:1000), anti-pSer15 p53 (Cell Signaling, #9286, 1:250), anti-p53 (DO-1, Santa Cruz, sc-126, 1:500) anti-Chk2 (H-300, Santa Cruz, sc-9064, 1:500), anti-pThr68 Chk2 (Cell Signaling, #2661, 1:500), anti-pSer1981 ATM (Cell Signaling, #4526, 1:500), anti-pSer317 Chk1 (Cell Signaling, #2344, 1:500), anti-Chk1 (Cell Signalling, #2360, 1:500), anti-pSer428 ATR (Cell Signalling, #2853, 1:500), anti-ATR (sc-1887, Santa Cruz, 1:500), anti-α-Tubulin (Sigma, T5168, 1:10,000), anti-p21 (sc-397, Santa Cruz, 1:250), anti-CREST (CS1058, Cortex Biochem, 1:5000). The following secondary antibodies were used for western blot experiments: peroxidase-conjugated goat anti-rabbit (P448 DAKO, 1:2000) and goat anti-mouse (P447 DAKO, 1:2000). Secondary antibodies used for immunofluorescence and FACS analysis were anti-rabbit Alexa 488 (A11008 Molecular probes, 1:600), anti-mouse Alexa 568 (A11004 Molecular probes, 1:600). DAPI was used at a final concentration of 1 μg/ml.

### Senescence-associated beta-galactosidase (SA-β-gal) activity assays

To detect SA-β-gal by cytochemistry, cells were fixed for 5 min using 2% formaldehyde 0,2% gluteraldehyde in PBS after 6 days of gRNA transfection. Cells were washed three times with PBS before overnight (16 h) incubation in staining solution (X-gal in dimethylformamide (1 mg ml), citric acid/sodium phosphate buffer at pH6 (40 mM), potassium ferrocyanide (5 mM), potassium ferricyanide (5 mM), sodium chloride (150 mM) and magnesium chloride (2 mM)) at 37°C (not in a CO_2_ incubator). Cells were washed with PBS and the blue staining was detected using a CCD microscope equipped with a Zeiss AxioCam colour camera (Axiocam HRc). The C_12_FDG assay is based on the hydrolysis of a membrane permeable molecule, the 5-dodecanoylaminofluorescein di-β-d-galactopyranoside (C_12_FDG), by β-galactosidase enriched in senescent cells. After hydrolysis and laser excitation, the C_12_FDG emits green fluorescence and can therefore be detected by flow cytometry. After 6 days of gRNA transfection, RPE-1 were incubated with the Bafilomycin A1 solution for 1 h at 37°C, 5% CO_2_ followed by the C_12_FDG staining for 2 h at 37°C, 5% CO_2_. After incubation, RPE-1 were trypsinized and re-suspended with PBS. Cells were analyzed immediately using FACS Calibur flow cytometry. C_12_FDG was measured on the FL1 detector.

## RESULTS

### iCut is a tunable Cas9 system

**Figure 1.**
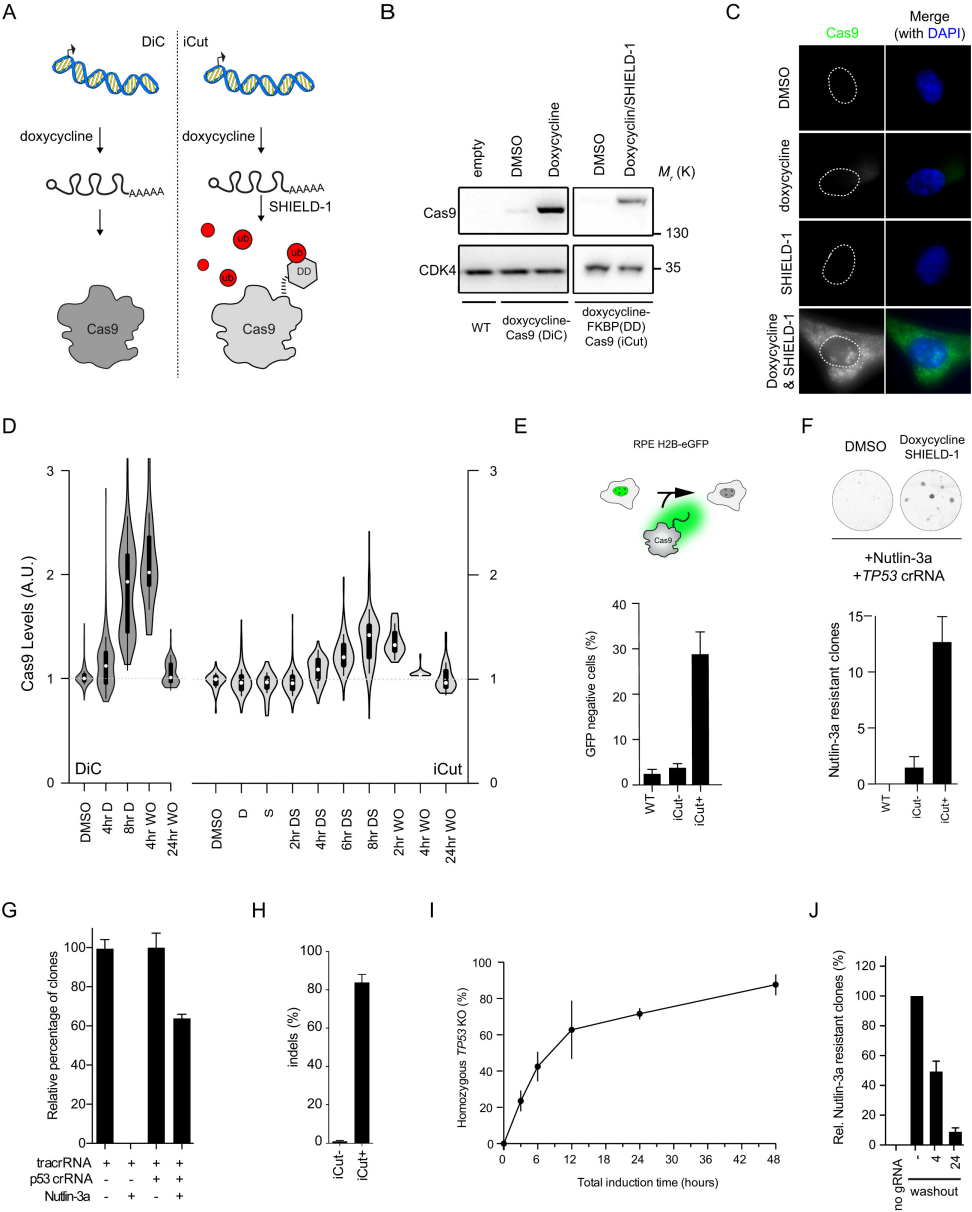
iCut is a tunable expression system for Cas9. (**A**) Schematic representation of iCut, where levels of Cas9 can be regulated on both transcriptional and protein level. (**B**) Western blot (WB) analysis of Cas9 expression level iCut cells after the addition of doxycycline and SHIELD-1. (**C**) Immunofluorescence (IF) of iCut cells treated with DMSO (–), doxycycline (D), SHIELD-1 (S) or both agonists (DS). (**D**) IF Quantification of Cas9 levels in DiC and iCut cells with indicated conditions. Washouts (WO) were performed after 8 h of induction with the respective agonists. Each condition contained a least 50 cells derived from three independent. (**E**) Top: Schematic representation of H2B-eGFP assay. Bottom - Targeting of H2B-eGFP RPE-1 cells with iCut, DS was added for 8 h following rigorous washout. Cells were allowed to recover for 4 days. eGFP+ was determined by flow cytometry (Supplemental 2B, C). The average was determined from three independent experiments (error bars represent SEM).( **F**) Top: Clonogenic assay for *TP53* edited iCut cells. Agonists were added for 8 h following rigorous washout, cells were allowed to recover for 24 h. Subsequently, 250 cells were plated and selected with 10 μM of Nutlin-3a. Bottom: Quantification of the number of Nutlin-3a selected clones to grow out in the different conditions. (**G**) The absolute efficiency achieved with our system by comparing Nutlin-3a with DMSO in both edited and unedited iCut cells. Agonists were added for 24 h. The average was determined from three independent experiments (error bars represent SEM).( **H**) The number of insertions and deletions determined by TIDE of the edited and unedited cells prior to selection. The average was determined from three independent experiments (error bars represent SD). (**I**) Time-course of iCut activity in the presence of p53 gRNA, treated similarly to Figure 2D, *TP53KO* (%) is a ratio of Nutlin-3a/DMSO clonal outgrowth. The average was determined from three independent experiments (error bars represent SD).( **J**) Reversibility assay of iCut system. Cells were transfected at indicated times with p53 gRNA and subsequently selected with Nutlin-3a. Timepoints indicate period after washout of doxycycline and SHIELD-1. Outgrowth was normalized to constitutive activation (–washout, error bars represent SD).

Genome editing tools allow one to introduce DNA breaks at well-defined sites. In theory, this makes it possible to introduce a defined number of breaks in a single genome. But to render such a nuclease-based system comparable to classical DNA damaging agents it is essential to obtain temporal control over nuclease activity to prevent a cycle of cut and repair. To this end, we brought Cas9 under control of a 3^rd^ generation doxycycline-inducible promoter (23) and introduced this into RPE-1 cells, a human diploid retinal pigment epithelial cell line immortalized by ectopic expression of hTERT (34). While doxycycline could induce high levels of expression of Cas9 (DiC-RPE-1), we also observed considerable expression of Cas9 in the absence of doxycycline (Figure 1B), making the system not suitable to use for time-controlled break formation. Therefore, we decided to add a destabilization domain (FKBP-degron) to the N-terminus of Cas9 and put this construct under the control of the same doxycycline-inducible promoter (Figure 1A) (30,35). Importantly, the doxycycline-regulated FKBP-tagged Cas9 (iCut) was not expressed at detectable levels in the iCut-containing RPE-1 (iCut-RPE-1) cells treated with DMSO only (Figure 1B). The addition of doxycycline (D) to the iCut-RPE-1 cells did not lead to low levels of Cas9 expression, while the combination of doxycycline and SHIELD-1 (DS) induced high levels of Cas9 expression (Figure 1B–D). Following rigorous washout, the levels of Cas9 in iCut-RPE-1 rapidly decreased (Figure 1D). Four hours after washout, levels of expression were still near maximal in the DiC-RPE-1 cells, while they were close to basal levels in the iCut-RPE-1 cells (Figure 1D). Additionally, we confirmed that iCut activation alone does not result in altered cell cycle distribution or proliferative defects (Supplementary Figure S1A,B). Taken together, in iCut-RPE-1 cells, Cas9 can be switched on and off in a time-controlled manner.

### Highly efficient gene inactivation with iCut

To functionally validate the iCut system, we first introduced a single copy of H2B-eGFP (36) into the iCut-RPE-1 cells by lentiviral transduction (Figure 1E). Subsequently, we introduced a guide RNA (gRNA) targeting the transgene and transiently activated Cas9 using the appropriate agonists for 8 h followed by rigorous washout. Subsequently, we allowed the cells to recover for four days to allow for H2B-eGFP protein turnover. In iCut cells, we observed a loss in GFP-expression in ∼30% of the cells (Figure 1E). The percentage of GFP-negative cells was higher in DiC-RPE-1 cells (∼60%), but we also found up to 40% GFP-negative cells in the non-induced DiC-RPE-1 cultures, consistent with the observation that Cas9 is expressed in these cells in the absence of doxycycline (Supplementary Figure S1C). In sharp contrast, there was no significant increase in the percentage of GFP-negative cells in the iCut-RPE-1 treated with DMSO (iCut-), indicating that Cas9 activity is absent in iCut-RPE-1 cells in the absence of agonists (Figure 1E). Next, we monitored genome editing-efficiency of the iCut system by exploiting a small molecule inhibitor for MDM2, Nutlin-3a. Nutlin-3a inhibits proliferation of p53-proficient cells but is ineffective in p53-deficient cells (37). Thus, only cells in which both alleles of *TP53* have been inactivated will proliferate in the presence of Nutlin3a. In accordance with previous results, we find that the highest mutation efficiency is obtained in the DiC-RPE-1 cells, but again, this system proved to be very leaky (Supplementary Figure S1D). Conversely, iCut cells display no editing activity in DMSO treated condition. However, iCut activity in the presence of doxycycline and SHIELD-1 practically reaches the levels of activated DiC-RPE-1 cells (Figure 1F).

In order to quantify the absolute activity of iCut, we transfected cells with gRNA for *TP53* or control and activated iCut for 24 h. We allowed the cells to recover for 48 h and plated them for clonogenic assays in the presence or absence of Nutlin-3a. Approximately 60% of the cells subjected to Cas9-induced genome-editing became resistant to Nutlin-3a, indicating that both alleles of *TP53* were inactivated in this fraction of the population (Figure 1G). Application of the TIDE assay (26) was performed in parallel, which showed that as much as 80% of the alleles had been edited in the absence of Nutlin-3a selection (Figure 1H), all of them resulting in frame-shift mutations (Supplementary Figure S1E, F). We could confirm that the editing efficiency is dependent on the time iCut is activated (Figure 1I). iCut activation for 3 h results in ∼25% Nutlin-3a-resistant cells. After 6 h about half of the cells were able to grow out in Nutlin-3a, while at 12 h of activation approximately two-thirds of the cells had become resistant. Longer incubation with the agonists led to a slow but consistent increase in overall genome-editing (Figure 1I), indicating that we reach near-maximum genome editing capacity after 12 h of agonist addition. As an additional quality control for the iCut system, we set out to test reversibility of genome editing capacity following washout of the agonists. We first activated the DiC or iCut system for 16 h and subsequently washed out the agonists. At 4 or 24 h following the washout of agonists we transfected the p53- or H2B-GFP-targeting gRNAs and checked genome-editing capacity. As expected, washout of doxycycline from the DiC-RPE-1 cells did not result in a decrease in activity towards *TP53* (Supplementary Figure S1G). In contrast, washout of doxycycline/SHIELD-1 led to a sharp decline in genome editing over time (Figure 1J). Taken together, we have generated a tagged-Cas9 that allows us to regulate genome editing capacity over time.

### Introducing a defined number of breaks with iCut

**Figure 2.**
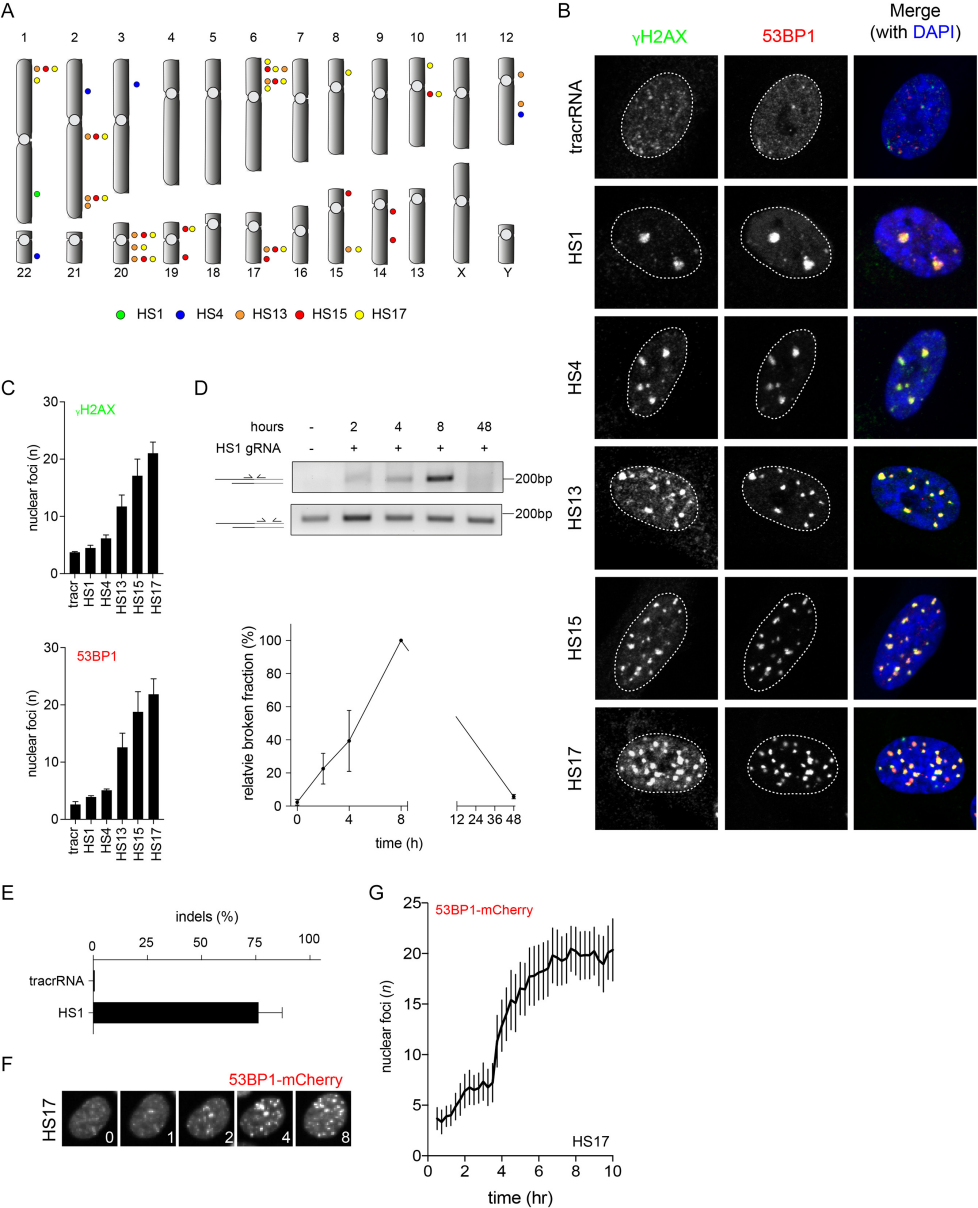
Introducing a defined number of breaks with iCut. (**A**) Overview of genomic targeting locations with a set of gRNAs. (**B**) iCut cells were transfected with the corresponding gRNA,16 h later agonists were added for 8 h. Subsequently, cells were fixed and stained with γH2AX and 53BP1 to visualize DNA damage. (**C**) Quantification of the number of nuclear foci for γH2AX and 53BP1. The average was determined from three independent experiments consisting of at least 50 cells (error bars represent 95% CI). (**D**) Ligation mediated PCR on the HS1 locus with corresponding PCR primers (see Materials and Methods). Quantification of the broken normalized to the relative maximum broken DNA (8 h post transfection). (**E**) TIDE indel analysis 48 h following transfection of the HS1 gRNA. (**F**) Representative images from 53BP1-mCherry time-lapse of iCut cells transfected with HS17 G) Quantification of the number of 53BP1-mCherry foci straight after transfecting HS17 gRNA and traced DNA damage foci formation for a time period of 10 h (33 cells from three independent experiment, error bars represent 95% CI).

We next wanted to probe whether gRNAs that target multiple homologous sites can be used in the iCut system to introduce multiple DSBs. To this end, we generated gRNAs targeting multiple sequences in the genome, which according to the CRISPOR (27) prediction tool will target 1, 4, 13, 15 or 17 homologous sites (HS1, HS4, HS13, HS15 and HS17, respectively) (Supplementary Table S1, Figure 2A). All sites were selected based on their high on- and low off-target scores (see Materials and Methods). Using these gRNAs, we set out to investigate if the iCut system can be used to introduce an increasing number of DNA breaks. Activation of Cas9 by addition of doxycycline and SHIELD-1 occurred 16 h prior to transfection of the gRNA. Eight hours following transfection, we fixed the cells and monitored DNA damage-induced foci using canonical DNA damage markers: 53BP1 and phosphorylated H2AX (γH2AX). We find that the average number of γH2AX foci ranges from 4 to 21 foci, depending on the gRNA used, whereas we find on average three background foci in the tracr control (Figure 2B, C). A similar number of foci are detected with 53BP1 by this set of gRNAs (Figure 2B, C). Importantly, the number of breaks that we observed seem to nicely fit with the predicted number of target sites when taking into account that a subset of cells will be in G2 (4 versus 2 alleles) and the fact that not all target sites are cut within 8 h of activation. More accurately, we expect approximately half of the target sites to be broken around 8 h following (see Figure 1I); so the observed numbers of breaks approximate the expected number (Figure 2B,C). Additionally, we observed that Cas9-induced foci appeared relatively large, suggestive of extensive signaling at the break site. Therefore, we set out to investigate whether these foci were larger than γ-IR-induced foci. In order to trace the appearance of DSBs in living cells, we made use of a 53BP1-mCherry reporter. We either transfected cells with the HS1 gRNA or we irradiated iCut cells with 0.5 Gy and quantified 53BP1 retention time, maximum focus size and intensity. We observed that the maximum focus intensity does not differ between the two conditions (Supplementary Figure S2A), but that both maximum foci size and retention time were increased for the Cas9-induced break compared to IR-induced breaks (Supplementary Figure S2B, C). From these data we can conclude that CRISPR-induced breaks take on average ∼2 h longer to be repaired than IR-induced breaks, possibly as a consequence of the fact that Cas9 remains bound to broken DNA (38).

While γ-Irradiation results in an instantaneous accumulation of DSBs, the timing in which Cas9 is able to target multiple loci has not been established. Therefore, we set out to monitor the time it takes for Cas9 to generate DSBs after supplying the gRNA. We determined kinetics of break formation following gRNA transfection by means of ligation-mediated PCRs (Figure 2D) (30). Double strand breaks are detectable as early as 2 h after activation of the iCut system, and their incidence rises for a few hours post gRNA transfection (Figure 2D). Eight hours following transfection we observe the highest level of broken DNA, but at 48 h after transfection we find that these breaks are gone. This is also supported by data obtained by sequencing of the HS-target site, which show that ∼75% of the targeted locus has been edited after 48 h (Figure 2E). In order to validate the kinetics of break induction, we used iCut cells expressing 53BP1-mCherry and transfected them with the HS17 gRNA. We observe a sharp accumulation of DSBs (Figure 2F) at 4 h post-transfection, reaching maximum break formation around 6 h (Figure 2G). Taken together, we are able to induce a well-defined number of DNA breaks at defined locations in single iCut RPE-1 cells with high efficiency and specificity with a sharp time-resolution.

### A single break is sufficient to activate the DNA damage checkpoint

**Figure 3.**
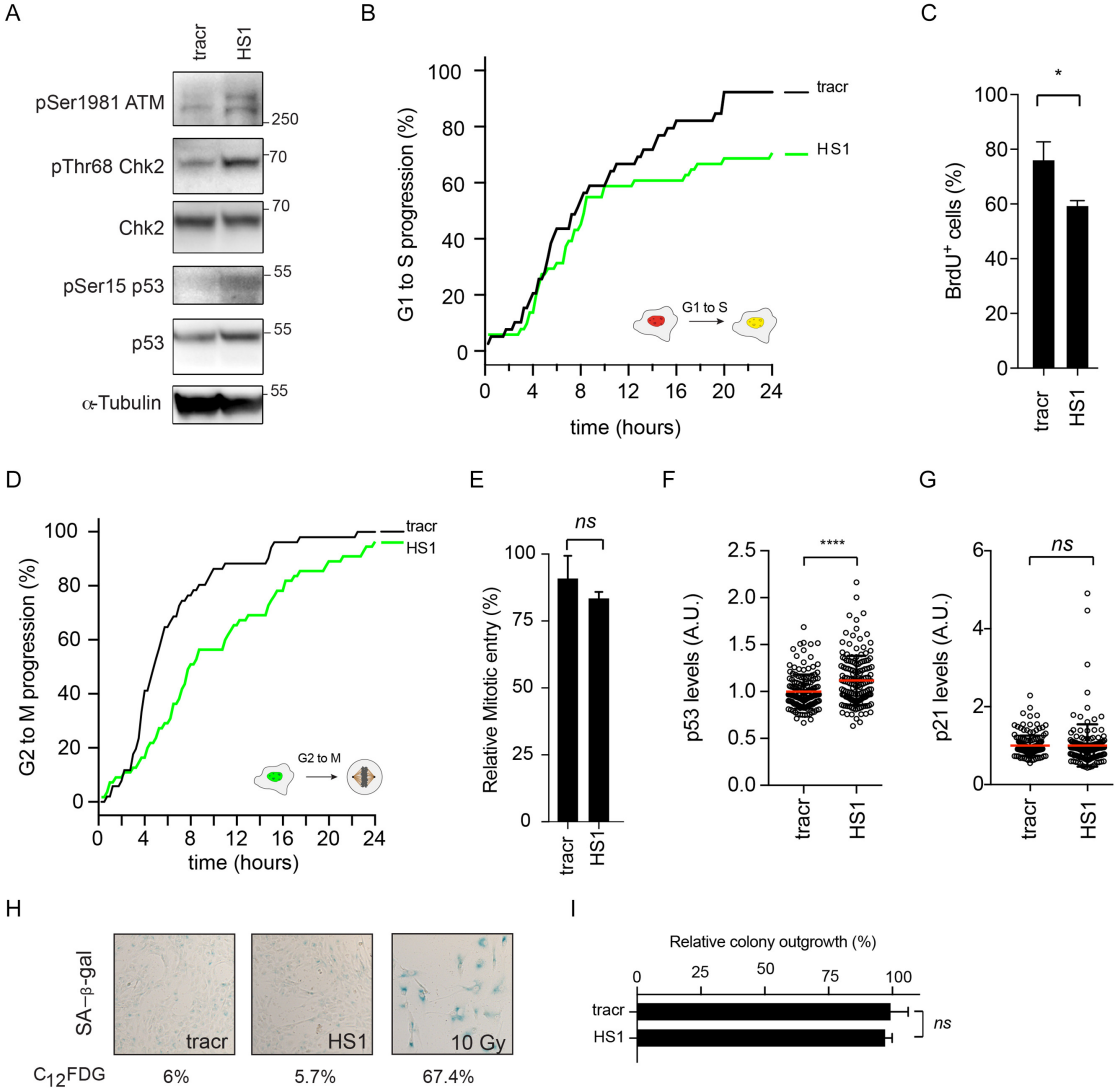
A single breaks is sufficient to activate the DNA damage checkpoint. (**A**) Western Blot analysis of DNA damage checkpoint signaling. (**B**) Live cell imaging of RPE-1 FUCCI iCut cells. Cumulative S-phase entry over 24 h of RPE-1 iCut FUCCI cells challenged with indicated gRNAs. S-phase entry was quantified starting 8 h following transfection and was determined from three independent experiments consisting of at least 50 cells. (**C**) S-phase entry was determined by quantifying the number BrdU positive cells following sixteen our incubation. Student's *t*-test was performed to asses significance. (*P < 0.05).( **D**) Live cell imaging of RPE-1 FUCCI iCut cells - Cumulative mitotic entry over 24 h of RPE-1 iCut FUCCI cells treated with indicated conditions. Mitotic entry was quantified starting 8 h following transfection and was determined from three independent experiments consisting of at least 50 cells. (**E**) G2 arrest following 8 h of iCut induction in double-thymidine blocked cells with corresponding gRNA. Checkpoint recovery was assayed in the presence of Nocodazole for 16 h cells were stained with MPM-2 to determine the mitotic percentage. The average was determined from three independent experiments (error bars represent SD).( **F**) Quantitative-IF for p53 levels 8 h post transfected of indicated gRNAs. Student's *t*-test was performed to asses significance (****P < 0.0001). (**G**) Quantitative-IF for p21 levels 8 h post transfected of indicated gRNAs. Student's *t*-test was performed to asses significance (*ns* = not significant). (**H**) Senescence-associated β-galactosidase staining of RPE-1 hTERT. Cells were transfected with indicated gRNAs and allowed to recover for 6 days and subsequently stained. Percentages indicate flow cytometric assessment of C_12_FDG, a fluorescent analog of SA-β-Gal. (**I**) Clonogenic assay with 24 h of iCut induction with the corresponding gRNA and subsequent outgrowth for 7 days. Student's *t*-test was performed to asses significance (*ns* = not significant).

There is evidence to suggest that a single DSB is insufficient for proper checkpoint activation in mammalian cells (39,40). On the other hand, ectopic recruitment of 100–150 identical DNA damage recognition and checkpoint proteins to a single copy LacO array is sufficient to induce a potent G2 checkpoint (41). Additionally, a single HO nuclease-induced DSB in *Saccharomyces cerevisiae* is able to block cells from entering mitosis, indicating that a single DSB is sufficient in yeast to induce and maintain a G2 arrest (42). These data do not combine into a gratifying perspective on the capabilities and limitations of DNA damage checkpoints. Using the iCut system, we set out to investigate checkpoint activation at a low number of double-strand breaks. To visualize checkpoint activation, we harvest cells 8 h after transfection and investigate the levels of phospho-Ser1981 ATM and phospho-Thr68 on Chk2, a canonical ATM target (43). The activated Chk2 kinase will, in turn, phosphorylate p53 on Ser15/20, allowing it to activate its transcriptional targets, such as the Cdk-inhibitor p21 (44–46). Interestingly, we observe a clear induction of DNA damage signaling after the introduction of a gRNA targeting a single site (HS1) in the human genome. This is evidenced by increased phosphorylation of Ser1981 on ATM, Thr68 on Chk2 and Ser15 on p53 (Figure 3A). Thus, low levels of DNA damage (1–4 breaks) are able to induce ATM, Chk2 and p53 phosphorylation (Figure 3A). Besides the ATM-Chk2 signaling axis, we wondered whether a limited number of CRISPR-induced breaks would be sufficient to activate the ATR-Chk1 signaling axis. In order to address this, we synchronized iCut cells in G2 and transfected HS1 gRNA or tracr control. We observed an increase in both ATR activity (pSer428 ATR) and Chk1 activity (pSer317 Chk1) (Supplementary Figure S3A). This suggests that these breaks are also templates for resection-dependent repair. In order to investigate this further, we employed TIDE (26) indel sequencing on G2 synchronized cells. Indeed, we found that approximately 30% of the repair products were the result of classical NHEJ (Supplementary Figure S3B). These repair products are represented by small deletions (+1, +2, –1, –2, –3, –4) that are also formed when end-resection is blocked using the Mre11 inhibitor Mirin (Supplementary Figure S3B), indicating that these must be repaired via NHEJ. Consistent with this notion, these repair products are reduced when DNA-PKcs is inhibited, confirming that they are formed through NHEJ (Supplementary Figure S3B). The other class of repair products required active resection (–5, –8) (Supplementary Figure S3B) and their appearance was strongly reduced by addition of Mirin, confirming that these do depend on end-resection (Supplementary Figure S3B). Thus, repair of CRISPR-induced breaks can occur through resection-dependent and resection-independent pathways, and both occur relatively efficiently.

Since we observe mild but reproducible checkpoint activation with a low amount of DSBs, we next investigated how the introduction of low levels of DSBs would affect cell cycle progression. Using live cell imaging of RPE-1 FUCCI cells with iCut (Figure 3B, D); we determined the cell cycle transitions, from G1 to S and G2 to M, after introducing the HS1 gRNA. In G1 cells, we find a drop in S phase entry around 10 h after induction of break formation. Given the fact that it takes ∼4–6 h for breaks to form, this suggests that the late G1 cells that have passed the restriction point fail to arrest, while the early G1 cells do arrest in response to 1 or 2 breaks (Figure 3B). Overall, the response to HS1 results in ∼20% less S-phase entry over a time period of 24 h (Figure 3B). We were able to confirm the decrease in S-phase entry following low-level DNA damage by performing BrdU incorporation (Figure 3C). In G2 cells, we see that mitotic entry is reduced at as little as 2 h after break induction (Figure 3D). Introduction of HS1 resulted in a delay in mitotic entry of about 4–6 h, but eventually, most of the cells entered mitosis (Figure 3D). To further corroborate these findings, DSBs were introduced in G2-synchronized cells, after which we determined the ability of cells to enter mitosis. No significant decrease in the fraction of cells entering mitosis could be observed in cells containing HS1 gRNA (Figure 3E).

To investigate whether the HS1-induced cell cycle arrest can result in a permanent withdrawal from the cell cycle, we first out to investigate the upregulation of p53 in response to a gRNA targeting a single site. Induction of a DNA damage-induced arrest is associated with stabilization of p53, both in G1 and in G2 (45–47). Using immunofluorescence, we could show that expression of p53 does increase after the generation of a single break (HS1) compared to tracr control (Figure 3F). Strikingly, we observed no upregulation of p21 in cells challenged with a single DSB (Figure 3G). This implies that the level of expression of p53 that is achieved after introduction of 1–4 breaks is too low to promote transcriptional upregulation of p21 required to drive cells out of cycle (32,48). To address this directly, we quantified the fraction of senescent cells seven days following the initial DNA damage insult. Cells treated with HS1 did not display an increased propensity to enter senescence (SA-β-Gal, Figure 3H). We were able to validate these results by means of flow cytometric analysis of C_12_FDG, a fluorescent analog of β-Gal (Figure 3H and Supplementary Figure S3C). Conversely, cells challenged with 10 Gy of irradiation readily converted to senescent cells (SA-β-Gal) and ∼70% of the cells became C_12_FDG positive (Figure 3H & Supplementary Figure S3C). To obtain independent confirmation that a limited number of breaks do not trigger a withdrawal from the cell cycle, we performed clonogenic assays to monitor the ability of cells to grow out after DSB formation. Using HS1, we find a minor decrease in colony outgrowth ∼5% compared to control (tracrRNA alone) cells, confirming this number of breaks is not enough to cause a significant proliferative disadvantage (Figure 3I). Irradiating cells with 10 Gy invariably led to zero colonies (data not shown), suggesting that formation of multiple breaks does cause cells to permanently withdraw from the proliferative cycle. To study if a similar response could be induced using our multi-cutting guide RNAs, we used our set of gRNAs to determine the overall responsiveness of RPE-1 cells to CRISPR-induced breaks, as compared to IR-induced breaks. Using a dose range up to 2 Gy of IR, resulted in a dose range of 0–20 double strand breaks, as determined by staining for 53BP1 and γH2AX foci (Supplementary Figure S3D). By means of FUCCI live cell imaging, we observed a graded decrease in G1 to S phase progression with increasing number of Cas9-induced breaks (Supplementary Figure S3E). However, G2 to M progression revealed a bimodal trend. While targeting HS1 and HS4 resulted in a modest decrease in mitotic entry, 80 to 90% of the cells targeted with HS13, HS15 and HS17 could not progress into mitosis (Figure 3C and Supplementary Figure S3E). This trend was further confirmed by analysis of mitotic entry using flow cytometry (Supplementary Figure S3F). Using 1–2 Gy of IR, which creates 10–20 DSBs (Supplementary Figure S3D), we find that mitotic entry is reduced to 50–80%, respectively (Supplementary Figure S3G). Thus, cells do seem to be somewhat more sensitive to Cas9-induced breaks, as compared to IR-induced breaks, when we assess the ability to mount a G2 checkpoint, possibly as a consequence of the fact that it takes more time to repair a Cas9-induced break (Supplementary Figure S2B). Consistent with this, we find that introduction of HS13-17 causes a more prominent activation of p53, as compared to HS1 or HS4, as well as in comparison to IR doses of 0-2 Gy (Supplementary Figure S3H). Additionally, when we challenge cells with HS13, HS15 or HS17 senescent cells appear in our population, but not with HS1 and HS4 (Supplementary Figure S3I and Figure 3H), again indicating that induction of a large number of Cas9-induced breaks can trigger a permanent cell cycle exit.

Taken together, we observe that DNA damage signaling is activated by Cas9-induced double strand breaks, and that targeting a single site in the genome is sufficient to elicit a checkpoint response. This signaling is enough to halt cells for a different period of time in the G1 versus the G2 phase of the cell cycle. However, the induced arrest did not lead to a permanent cell cycle exit, indicating that cells are able to respond to very low levels of genotoxic stress but cope with this without removing themselves from the proliferating population.

### Abrogation of a single DSB induced G2 checkpoint causes genomic instability

**Figure 4.**
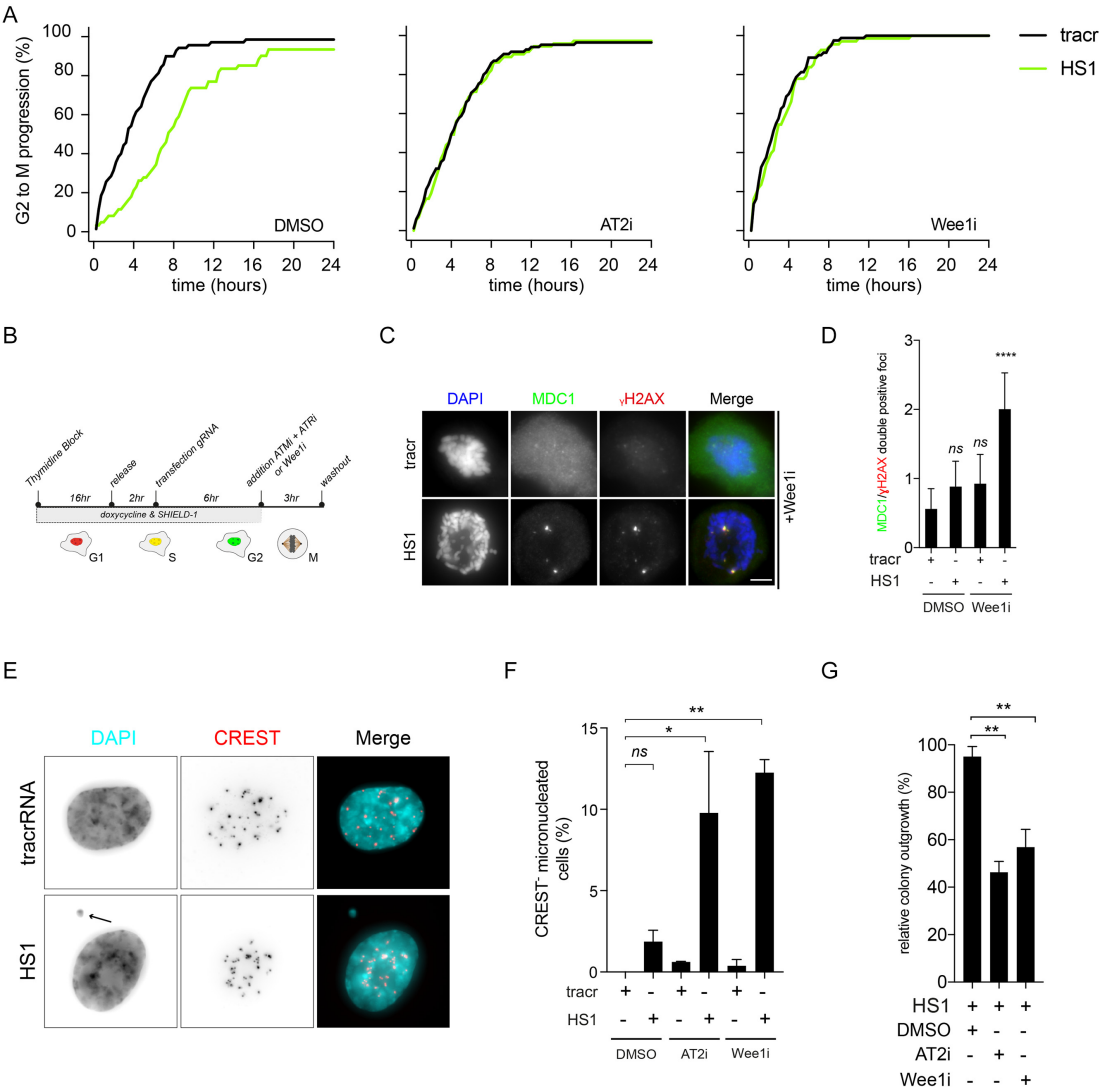
G2 checkpoint is essential to prevent genomic instability and maintain fitness in the presence of low numbers of breaks. (**A**) RPE-1 FUCCI iCut cells transfected with HS1 or tracr in the presence or absence of either AT2i (ATMi and ATRi) or Wee1i. Mitotic entry was quantified starting 8 h following transfection and was determined from three independent experiments consisting of at least 50 cells. (**B**) Schematic overview of G2 checkpoint ablation in the presence of low number of DNA breaks. (**C**) Nocodazole trapped mitotic cells transfected with HS1 or tracr in the presence of a Wee1i, stained for MDC1 and γH2AX. D) Quantification of Figure 4C. One way ANOVA with Bonferroni's multiple comparisons test was performed to asses significance (*ns* = not significant, ****P < 0.0001). (**E**) Nuclear morphology of cells treated with AT2i transfected with tracr or HS1. (**F**) Quantification of the phenotype observed in Figure 4E. One way ANOVA with Bonferroni's multiple comparisons test was performed to asses significance (*ns* = not significant, *P < 0.05. **P < 0.005). (**G**) Clonogenic outgrowth of cells transfected with HS1 normalized by tracr alone in corresponding conditions. One way ANOVA with Bonferroni's multiple comparisons test was performed to asses significance (*ns* = not significant, **P < 0.005).

It has been extensively shown that cells readily enter into mitosis in the presence of a low number of breaks (39,49,50). Therefore, our observation that cells display a checkpoint-induced delay in G2 in response to a low number of DSBs is quite unexpected and contradicts the current consensus in the field, stating that the G2 checkpoint is incapable of sensing a low number of breaks. However, our data thus far do not resolve if the delay is functional. In other words, is the delay required to allow time for DNA damage responses and faithful repair to occur? To address this, we asked if an override of this checkpoint response would comprise genomic integrity. To abrogate the activity of the G2 checkpoint we used two different settings, dual inhibition of ATM and ATR (AT2i) or inhibition of Wee1 (Wee1i). We used RPE-1 iCut FUCCI cells to trace single cells in which we assessed mitotic entry following break induction using HS1. Again, we observed a delay of ∼4–6 h in the HS1-transfected cells compared to tracr-transfected cells (Figure 4A, DMSO). Conversely, when we treated cells with either AT2i or Wee1i, the delay was drastically shortened (Figure 4A, AT2i & Wee1i). We wondered whether this checkpoint was also dependent on the described role of p53 to maintain a G2 cell cycle arrest (47). Therefore, we used RPE-1 iCut FUCCI *TP53Δ* cells transfected with HS1 in the absence or presence of inhibitors of ATM & ATR (AT2i) and quantified cumulative mitotic entry. We found that the delay is not dependent on p53 activity as it is not different from wild-type cells (Supplementary Figure S4A). However, the delay in *TP53Δ* cells remains dependent on the combined activity of the ATM and ATR kinases as previously observed in wild-type cells (Supplementary Figure S4A and Figure 4A). This implies that the delay is independent of the maintenance branch of DNA damage checkpoint that is mediated via p53-p21 signaling (47). This suggest that this delay is dependent on checkpoint-dependent inactivation of the cdc25 phosphatases (51). Next, we asked if this checkpoint override will cause cells to enter mitosis with residual DNA breaks (Figure 4B). Indeed, upon abrogation of the G2 checkpoint, HS1-transfected cells that had entered mitosis stained positive for γH2AX and MDC1 foci (Figure 4C, D). This indicates that the G2 checkpoint is required to prevent cells from entering mitosis, even if they only have a very limited number of breaks. Next, we wondered what the consequence is for cells when we ablate the HS1-induced G2 checkpoint. To our surprise, we observed a 5-fold increase in the frequency of micronuclei generated after overriding the G2 checkpoint by either AT2i or Wee1i (Figure 4E,F). This indicates that the checkpoint response in G2, triggered by 1–4 breaks is essential to prevent cell division in the presence of a broken chromosome. Since it was previously reported that the inheritance of a broken chromosome can severely affect cell viability (52), we subsequently analyzed the proliferative capacity of G2 synchronized HS1-treated cells with or without a functional G2 checkpoint. We find that in cells with a functional G2 checkpoint, viability is not affected, as evidenced by clonogenic outgrowth (Figure 4G). Consistent with our observation that HS1-transfected cells treated with AT2i or Wee1i slip into mitosis with DNA breaks, ablation of the G2 checkpoint hampers proliferation and outgrowth in HS1-transfected cells (Figure 4G). In order to put these data in a broader perspective, we used 0.16 Gy of γ-IR by which we generated 1–4 breaks (Supplementary Figure S4B,C). We validated that this number of DSBs would delay cells in G2 and would be abrogated by addition of Wee1 or ATM plus ATR (AT2i) inhibitors (Supplementary Figure S4D). As expected, G2 cells challenged with this dose of γ-IR without the ability to mount a checkpoint (Wee1i) present with higher levels of DSBs in the following mitosis (Supplementary Figure S4E). Additionally, these cells displayed increased levels of micronucleation and had reduced clonogenic outgrowth capacity (Supplementary Figure S4F,G).Taken together, a low number of breaks in G2 will activate a checkpoint response that is absolutely required to prevent the propagation of DNA lesions into mitosis and the subsequent generation of micronucleated cells, a hallmark of genomic instability.

## DISCUSSION

Here we describe that, by using a tractable Cas9 system, single double strand breaks are sufficient to induce a cell cycle arrest in both G1 and G2. Additionally, when we override this checkpoint in G2, cells progress through mitosis with unresolved DNA damage. This leads the generation of micro-nucleated cells and the loss of genome integrity. Intriguingly, these data imply that cells in G1 respond differently to a single DSB when compared to cells in G2. When the damage is encountered in G1, cells readily enter S-phase up to 10 h following DSB formation. After this period, inactivation of the G1 checkpoint is hindered as only a couple of cells are still able to enter S-phase beyond this point in time. Thus, cells that are in late G1 are incapable of mounting a checkpoint that prevents S phase entry, whereas the early G1 cells can. This late G1 population has previously been described to be beyond the restriction point and therefore unable to arrest in G1 (53–55). Conversely, the G2 checkpoint reacts much more rapidly to a single DSB, by blocking the progression into mitosis as early as 2 h after break formation. However, in contrast to the prolonged G1 arrest, the G2 arrest that is triggered by single breaks is much more transient in nature. Over time, cumulative mitotic entry catches up to the unperturbed cells in the HS1-treated condition, indicating that (almost) all of the arrested cells can revert the cell cycle block in 4–6 h (Figure 3E). This is dramatically different when multiple breaks are inflicted in a G2 cell, as we find that this causes many, if not all, of the G2 cells to permanently withdraw from the cell cycle. Taken together, these data show that cells with a minimal amount of DNA breaks are capable to mount an effective checkpoint response. The type of response depends on which phase of the cell cycle the damage is encountered. The deterministic factors distinguishing an arrest (G1) versus a delay (G2) remain to be uncovered, but iCut allows us to rule out location or number as a possible explanation for this difference. Also, it should be noted that the prolonged arrest in G1 induced by a single site targeting guide RNA is induced by 1–2 breaks, while the reversible arrest in G2 is induced by 1–4 breaks.

Future studies are required to identify the contribution of a single break among multiple DSBs to checkpoint signaling and establishment. To investigate this, sensitive biosensors of checkpoint activation (such as the ATM and ATR activity probe (50)) should be employed. Work from the Lahav lab has shown that cell fate dictated by the DNA damage response is in part regulated by the manner in which p53 transcriptional activity is induced and maintained over time (56,57). Recent work from this group showed that p53 levels need to rise above a threshold to execute apoptosis, but that this threshold increases with time after initial break formation (58). Thus, in order to induce a reversible cell cycle arrest, it is of the utmost importance to strictly keep p53 activity below the threshold required to induce apoptosis (58) or senescence (32,48,49). We find that this introduction of guide RNAs that recognize 13 or more sites in the genome supersedes this threshold and causes many cells to enter a senescent state. In light of results described here, it would be interesting to investigate the relationship between DNA checkpoint activation at specific locations and cell fate decisions governed by p53 signaling. Moreover, persistent breaks could lead to a longer or higher induction of damage-induced transcription leading to different outcomes which might be dependent on break location. Thus, we expect that the outcome of the response depends on more than just numbers of breaks and will also be dependent on the ease with which breaks are repaired, but more work is required to resolve this issue.

Our findings indicate that a single DSB has the potential to mount a DNA damage checkpoint. Particularly, the G2 checkpoint in this setting displays interesting characteristics such as a delay dependent on DNA damage checkpoint kinases and a surprising impact on genomic stability when abrogated. In light of these findings, it would be intriguing to assess whether responses are regulated in another fashion once we target different regions in the genome. One could hypothesize that differences occur once DSB targeting occurs more distal or proximal with respect to the centromere. Another exciting line of investigating would be to target DSBs in a region with high or low transcriptional activity. These options are now feasible by using CRISPR/Cas9 mediated breaks.

The checkpoint activity in response to a single break has considerable implications for genome-editing technologies. For example, cell cycle delays generated by Cas9 could be interpreted as in genome-wide screens or competition assays. This is illustrated by multiple papers recently published describing the negative effect on proliferation by targeting genes present in amplified regions of the genome (59,60). Moreover, the Bassik lab recently found higher performance of their genome-wide CRISPR screens by using safe-target gRNAs (61). In short, the control set of gRNAs used now generate a DSB, whereas previous non-targeting guideRNA did not. These findings underline our main conclusion that a limited number of breaks is sufficient to trigger a DNA damage response.

Recent data suggest CRISPR editing might be blocked by p53 transcriptional activity (62,63). Although we observe stabilization of p53 in our experiments, we do not observe a significant decrease in proliferation in wild-type cells, suggesting lack of p53 activity in response to limited number of CRISPR breaks. Furthermore, we observe a similar cell-cycle delay in TP53Δ cells, which can be rescued by inhibition of ATM and ATR kinases. Based on our data, it seems rather unlikely that in our system p53 directly influences cell fate when a single site is targeted, and would thereby limit CRISPR efficacy. An alternative explanation for the absence of a p53-induced cell-cycle effect might be related to the toxicity of the delivery method of Cas9 and gRNA, as a recent study suggests dramatically different stress responses towards delivery methods in p53 proficient hematopoietic stem cells (64). Our data suggests that p53 does not directly impede CRISPR editing in cells when a single site is targeted, but this is likely different when multiple sites are hit (either on- or off-target), as evidenced by our finding that HS13 and above are potent inducers of cellular senescence.

Finally, the DNA repair and damage response fields have generated many reporter systems based on integrating an I-SceI site into the genome couple to a functional read-out (65). These readouts vary from assaying DNA damage-induced inhibition of transcription in neighboring genes to quantifying the relative rate of either HR or NHEJ repair pathway (15,66,67). Initially, these systems were utilized to validate novel players in these responses. We would like to propose using this CRISPR system to induce DSBs at specific locations to determine the contribution of DNA sequence and chromatin in DDR and repair phenotypes.

## Supplementary Material

Supplementary Data
