# Supplementary material for: A limited number of double-strand DNA breaks is sufficient to delay cell cycle progression
Source: Nucleic Acids Res. 2018 Sep 3;46(19):10132–44. doi: 10.1093/nar/gky786 (PMC6212793; doi:10.1093/nar/gky786)
Supplement: Supplementary Data [file gky786_supplemental_files.zip › NAR-01497-D-2018.R1_Supplementary figure legends.pdf]

Supplementary figure legends

Supplemental Figure 1

A) Cell cycle profile of RPE-1 WT, RPE-1 DiC and RPE-1 iCut analyzed by Flow Cytometry. B) Growth curve of RPE-1 WT, DiC and iCut cells in the presence of agonists. C) eGFP-assisted Cas9 activity assay with constitutive Cas9 expression of DiC cells with or without agonists. D) TP53/Nutlin-3a assisted Cas9 activity assay with constitutive Cas9 expression or DiC cells with or without agonists. E) Indel spectrum as readout with TIDE in iCut cells (26). F) Top - Ten monoclonal *TP53* knockout lines analyzed on protein level derived from iCut cells. Bottom – Mutations in *TP53* targeted RPE-1 iCut cells G) Quantification of the number of Nutlin-3a selected clones to grow out in the RPE-1 DiC cell line. The average was determined from three independent experiments (error bars represent SEM).

Supplemental Figure 2

A) Analysis of 53BP1 focus intensity of either Cas9 induced (HS1) or irradiation (0.5Gy) in 53BP1-mCherry expressing iCut cells (*ns* = not significant). B) 53BP1 focus size of the foci quantified in Suppl. Fig. 3A. (**, p<0.005). C) Retention times of the foci quantified Suppl. Fig. 3A (**, p<0.005) For all three parameter (Suppl. Fig. 2A-C), we used a student T-test to assess significance.

Supplemental Figure 3

A) Western Blot analysis of ATR-Chk1 and ATM-Chk2 signaling axis for HS1 gRNA. B) Composition of insertions and deletion with HS1 gRNA treated with DMSO, NU7441 (1μM), mirin (500μM) (error bars represent SD). C) Cells were labeled with C_{12}FDG 6 days after treatment or transfection and analyzed by Flow Cytometry. Cells were irradiated with 10 Gy (Pink), transfected with the tracr (Grey) or HS1 (Green). D) Quantification of the number of nuclear foci for γH2AX and 53BP1 irradiated with the indicated dose of γ-IR. The average was determined from three independent experiments consisting of at least 50 cells (error bars represent 95% CI). . E) Live cell imaging of RPE-1 FUCCI iCut cells – Cumulative S-phase and mitotic entry over 24hours of RPE-1 iCut FUCCI cells treated with indicated gRNAs. Entry was quantified starting 8 hours following transfection and was determined from three independent experiments consisting of at least 50 cells. Dotted lines are cumulative entries tracr and HS1 as shown in Fig. 3B, D F) G2 arrest following 8hrs of iCut induction in double-thymidine blocked cells with corresponding gRNA. Checkpoint recovery was assayed in the presence of Nocodazole for 16hrs cells were stained with MPM-2 to determine the mitotic percentage. The average was determined from three independent experiments (error bars represent SD) G) G2 arrest of double-thymidine blocked cells with corresponding doses of irradiation. Checkpoint recovery was assayed in the presence of Nocodazole for 16hrs cells were stained with MPM-2 to determine the mitotic percentage. The average was determined from three independent

experiments (error bars represent SD) H) Left - Western Blot analysis of ATM-Chk2-p53-p21 signaling axis for HS1-HS17. Right – Cells were lysed 1hr after treatment with the indicated dose of irradiation. I) SA-β-galactosidase and C12FDG staining for indicated gRNAs.

Supplemental Figure 4

A) RPE-1 FUCCI TP53Δ iCut cells transfected with HS1 or tracr in the presence or absence of AT2i (ATMi and ATRi). Mitotic entry was quantified starting 8 hours following transfection and was determined from three independent experiments consisting of at least 50 cells. B) RPE-1 hTERT cells were treated with 0 and 0.16Gy of irradiation and were fixed 30 minutes later and stained with γH2AX and 53BP1 to visualize DNA damage. C) Quantification of the number of nuclear foci for γH2AX and 53BP1. The average was determined from three independent experiments consisting of at least 50 cells (error bars represent 95% CI). D) RPE-1 FUCCI cells irradiated with 0.16Gy or mock IR in the presence or absence of either AT2i (ATMi and ATRi) or Wee1i. Mitotic entry was quantified starting 8 hours following transfection and was determined from three independent experiments consisting of at least 50 cells. E) Quantification of MDC1 and γH2AX double positive foci in nocodazole trapped mitotic cells irradiated with 0.16Gy or 0Gy in the presence of a Wee1i. One way ANOVA with Bonferroni's multiple comparisons test was performed to asses significance. (*ns* = not significant, **** = $p<0.0001$). F) Quantification of the micronucleation phenotype. One way ANOVA with Bonferroni's multiple comparisons test was performed to asses significance (*ns* = not significant, * = $p<0.05$. ** $p < 0.005$). G) Clonogenic outgrowth of cells irradiated with 0.16Gy normalized 0Gy in corresponding conditions. One way ANOVA with Bonferroni's multiple comparisons test was performed to asses significance. (*ns* = not significant, * = $p<0.05$)

Supplemental Table 1

A) Target sites for gRNA used in Figs. 3-4
