# Supplementary figures and images for: A limited number of double-strand DNA breaks is sufficient to delay cell cycle progression
Source: Nucleic Acids Res. 2018 Sep 3;46(19):10132–44. doi: 10.1093/nar/gky786 (PMC6212793; doi:10.1093/nar/gky786)

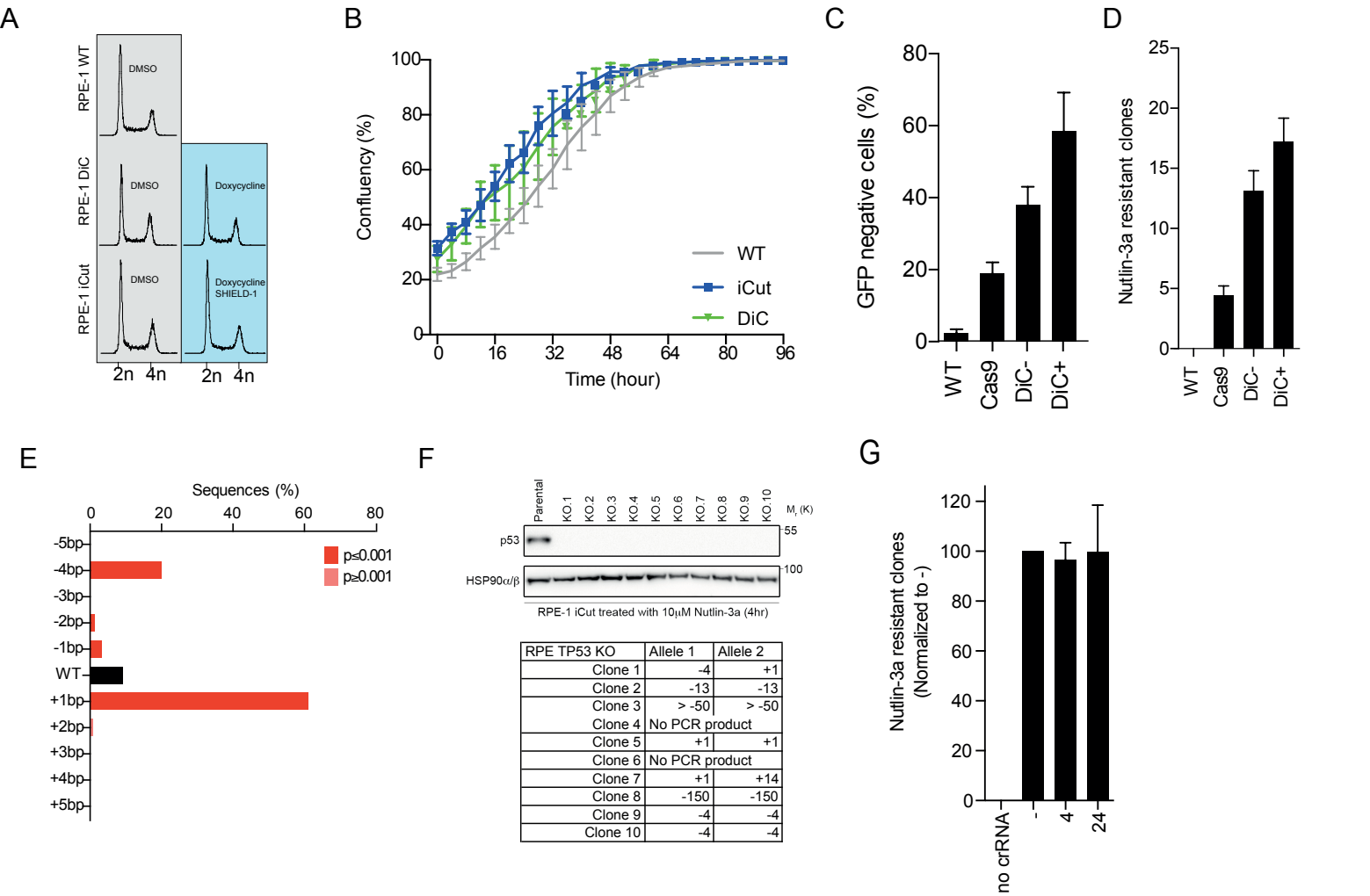

A

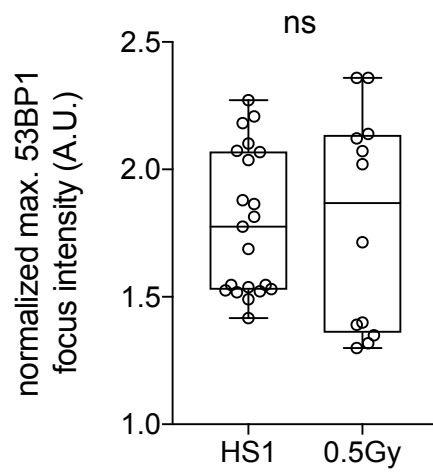

B

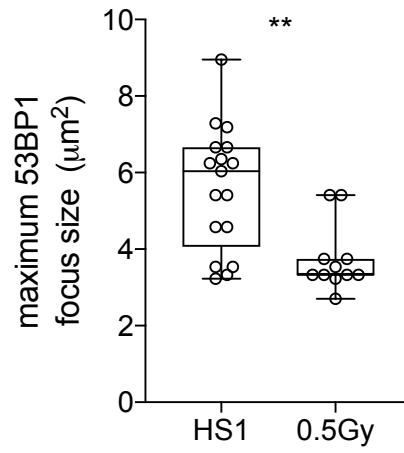

C

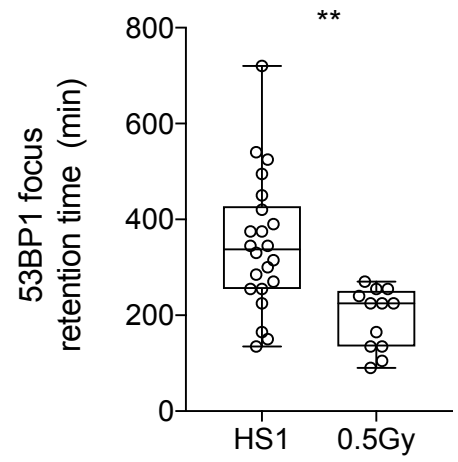

A

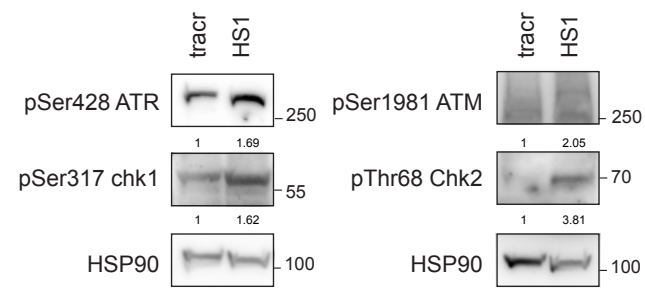

B

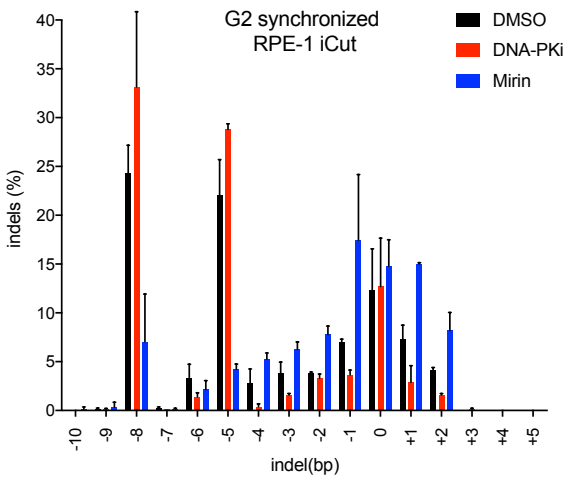

C

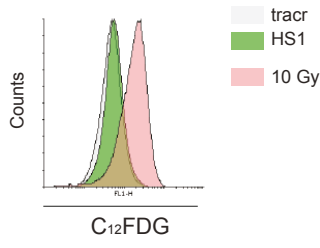

D

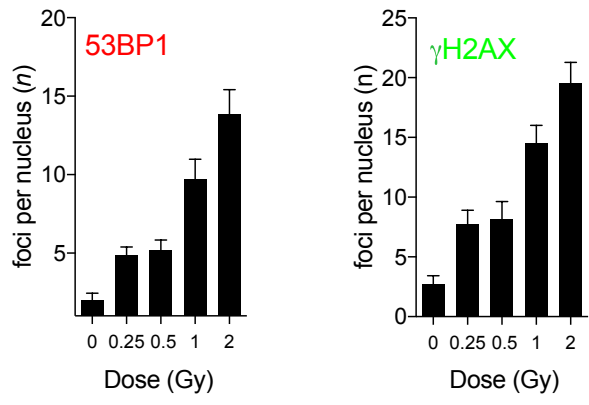

E

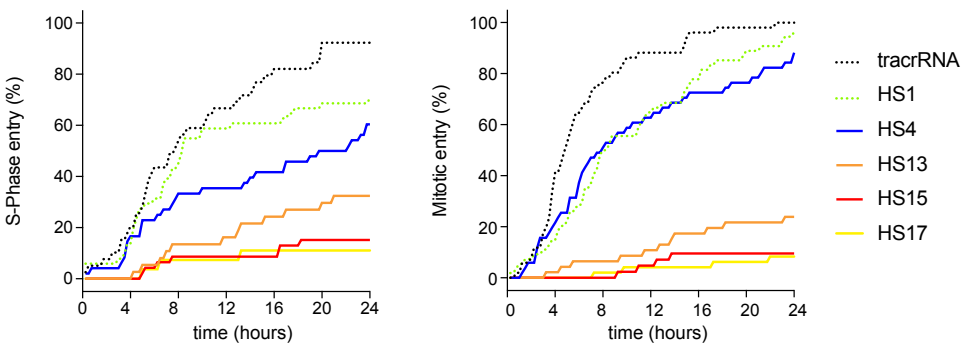

F

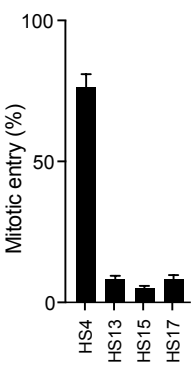

G

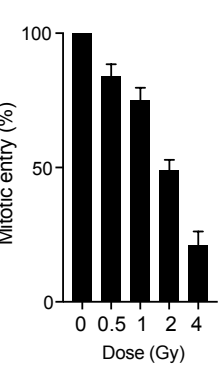

H

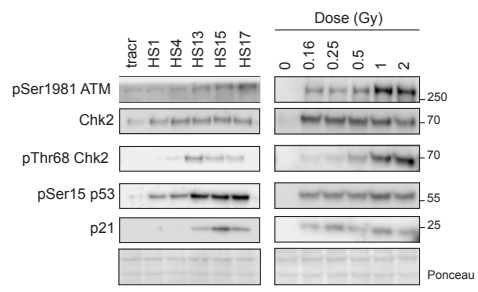

I

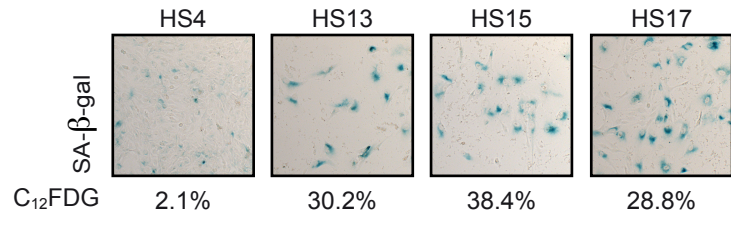

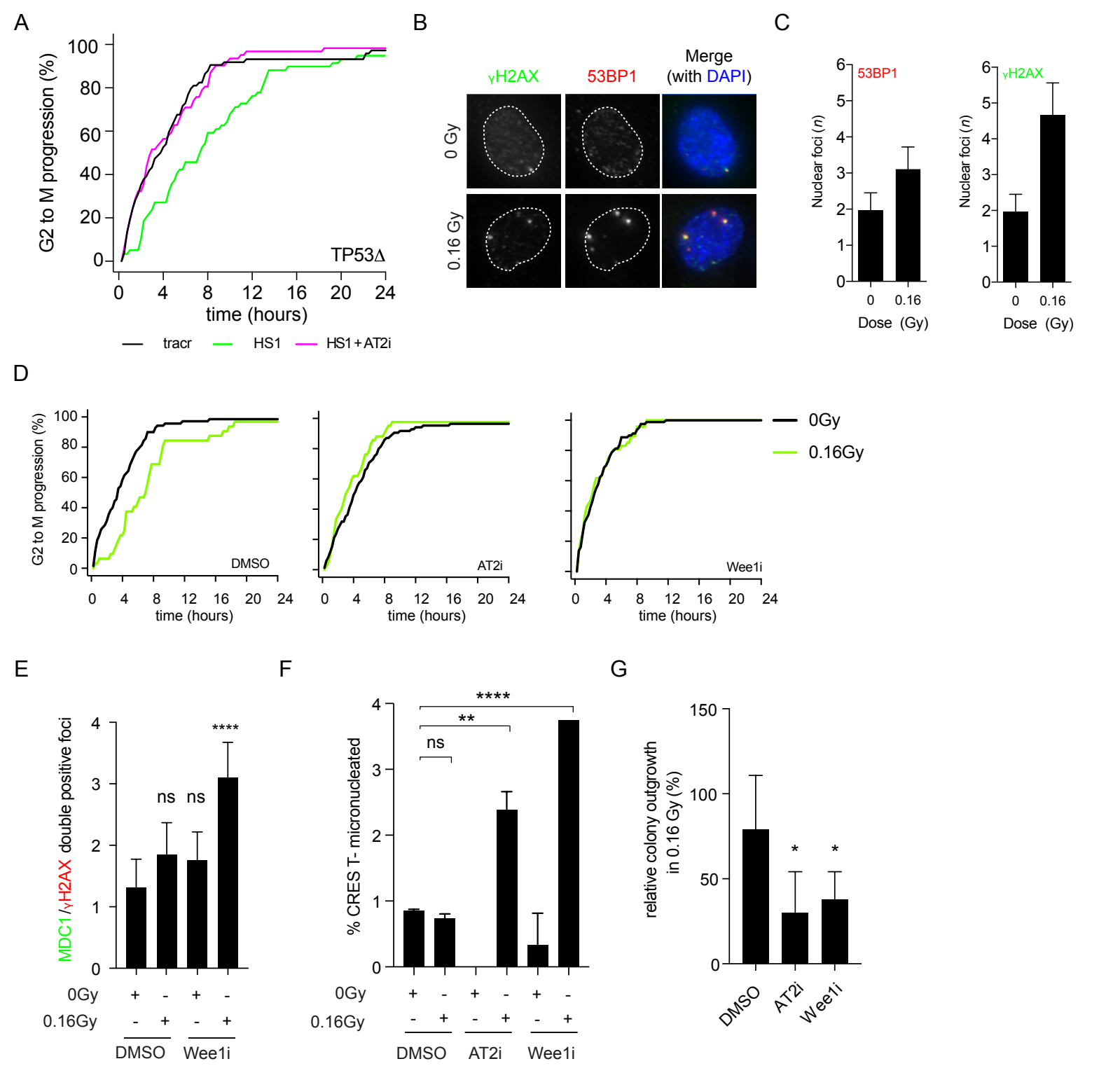

Supplement: Supplementary Data [file gky786_supplemental_files.zip › Supplemental_Figures.pdf]
